# Significance of genomic instability in breast cancer in atomic bomb survivors: analysis of microarray-comparative genomic hybridization

**DOI:** 10.1186/1748-717X-6-168

**Published:** 2011-12-07

**Authors:** Masahiro Oikawa, Koh-ichiro Yoshiura, Hisayoshi Kondo, Shiro Miura, Takeshi Nagayasu, Masahiro Nakashima

**Affiliations:** 1 Department of Human Genetics, Atomic Bomb Disease Institute, Nagasaki University Graduate School of Biomedical Sciences, Nagasaki, Japan; 2 Department of Surgical Oncology, Nagasaki University Graduate School of Biomedical Sciences, Nagasaki, Japan; 3 Biostatistics Section, Division of Scientific Data Registry, Atomic Bomb Disease Institute, Nagasaki University Graduate School of Biomedical Sciences, Nagasaki, Japan; 4 Tissue and Histopathology Section, Division of Scientific Data Registry, Atomic Bomb Disease Institute, Nagasaki University Graduate School of Biomedical Sciences, Nagasaki, Japan; 5 Department of Tumor and Diagnostic Pathology, Atomic Bomb Disease Institute, Nagasaki University Graduate School of Biomedical Sciences, Nagasaki, Japan

**Keywords:** breast cancer, atomic bomb survivors, radiation, genomic instability, CGH, microarray

## Abstract

**Background:**

It has been postulated that ionizing radiation induces breast cancers among atomic bomb (A-bomb) survivors. We have reported a higher incidence of *HER2 *and *C-MYC *oncogene amplification in breast cancers from A-bomb survivors. The purpose of this study was to clarify the effect of A-bomb radiation exposure on genomic instability (GIN), which is an important hallmark of carcinogenesis, in archival formalin-fixed paraffin-embedded (FFPE) tissues of breast cancer by using microarray-comparative genomic hybridization (aCGH).

**Methods:**

Tumor DNA was extracted from FFPE tissues of invasive ductal cancers from 15 survivors who were exposed at 1.5 km or less from the hypocenter and 13 calendar year-matched non-exposed patients followed by aCGH analysis using a high-density oligonucleotide microarray. The total length of copy number aberrations (CNA) was used as an indicator of GIN, and correlation with clinicopathological factors were statistically tested.

**Results:**

The mean of the derivative log ratio spread (DLRSpread), which estimates the noise by calculating the spread of log ratio differences between consecutive probes for all chromosomes, was 0.54 (range, 0.26 to 1.05). The concordance of results between aCGH and fluorescence in situ hybridization (FISH) for *HER2 *gene amplification was 88%. The incidence of *HER2 *amplification and histological grade was significantly higher in the A-bomb survivors than control group (P = 0.04, respectively). The total length of CNA tended to be larger in the A-bomb survivors (P = 0.15). Correlation analysis of CNA and clinicopathological factors revealed that DLRSpread was negatively correlated with that significantly (P = 0.034, r = -0.40). Multivariate analysis with covariance revealed that the exposure to A-bomb was a significant (P = 0.005) independent factor which was associated with larger total length of CNA of breast cancers.

**Conclusions:**

Thus, archival FFPE tissues from A-bomb survivors are useful for genome-wide aCGH analysis. Our results suggested that A-bomb radiation may affect the increased amount of CNA as a hallmark of GIN and, subsequently, be associated with a higher histologic grade in breast cancer found in A-bomb survivors.

## Background

Genomic instability (GIN) is an important hallmark of an enhanced carcinogenic process in human. Although there are various forms of GIN, many cancer cells show higher rates of chromosomal instability, which means changes in chromosome structure and number, compared with normal cells [1]. Recent cytogenetic analysis revealed that there were equal numbers of cytogenetic aberrations in solid cancers and hematological malignancies [2]. Several previous studies have reported the association between chromosome instability and GIN/clinical phenotypes in breast cancers. Fridlyand et al. [3] categorized three breast tumor subtypes based on copy number aberrations (CNA) in tumor DNA, which includes DNA copy number gains and losses, and suggested that these aberrations were related to shorter telomeres and the deregulation of the retinoblastoma (RB) gene pathway using an analysis of array comparative genomic hybridization (aCGH). Andre et al. [4] divided 106 breast cancers into three subtypes by the clustering method with the aCGH data and observed a correlation between cytogenetic subtypes and clinicopathologic characteristics, histological grade and intrinsic subtypes [5]. Hu et al. [6] and Melchor et al. [7] classified breast cancers by immunohistochemical staining pattern and found that triple-negative or basal-like subtype, which had the highest GIN among these subtypes, had the highest overall frequencies of CNA. Loo et al. [8] showed a correlation between fractional allelic loss and tumor size, mitotic rate and DNA content.

Atomic bomb (A-bomb) survivors who were exposed at young ages have already reached cancer-prone age. An increased risk of cancer has continued for decades, and the incidence of certain types of cancer is still higher in A-bomb survivors than in control populations [9-14]. It has been postulated that ionizing radiation induces breast cancers among A-bomb survivors. Our recent study demonstrated an association of *HER2 *and *C-MYC *oncogene amplification in breast cancers among A-bomb survivors with radiation exposure [15]. Oncogene amplification is thought to be associated with GIN and a main characteristic of solid tumors [16]. It is conceivable that radiation from the A-bomb 65 years ago may have induced a higher level of GIN in A-bomb survivors as a long-lasting health effect which is associated with the development of oncogene amplifications and subsequent carcinogenesis. However, the crucial mechanisms that can account for a radiation effect inducing GIN on the whole genome of breast cancers in A-bomb survivors remains elusive.

The rapid progress of technological innovation in biomedical science has enabled CGH analysis to be performed with higher resolution using high density oligonucleotide microarrays [17]. However, utilizing formalin-fixed paraffin-embedded (FFPE) archival tissue for the aCGH, which is the most common form of tissue preservation in routine practice, remains challenging. The main obstacle is DNA degradation, such as cross-linking between nucleic acid strands, DNA adducts with histones or nucleic acid binding proteins, and breaking and depurination of DNA. Recently, a one-step chemical labeling method, called the Universal Linkage System (ULS), has been put into production. This method yields precise, robust and high-quality aCGH data by labeling DNA with fluorescent dyes at the N7 position of guanine without enzymatic reaction, which is subject to perturbation by degraded DNA [18-20].

In the present study, we analyzed FFPE archival breast cancer tissues from A-bomb survivors by aCGH using a high density oligonucleotide microarray and ULS labeling to determine the effect of A-bomb radiation on genomic alterations during breast carcinogenesis. This study revealed a higher incidence of CNA in breast cancer tissue from A-bomb survivors than in tissue from calendar year-matched control patients, suggesting a role for GIN during breast carcinogenesis in A-bomb survivors. To the best of our knowledge, this is the first report of an aCGH analysis with solid tumors from A-bomb survivors.

## Methods

### Tumor samples and clinical information

All samples were FFPE tissues. An A-bomb survivor was defined in the present study as a person who received the "Atomic Bomb Survivor's Health Handbook" produced by Nagasaki city authorities since the establishment of the Atomic Bomb Survivors' Medical Treatment Law in April 1957. Our previous report has already identified 35 breast cancers from A-bomb survivors exposed at or less than 1.5 km from the hypocenter in pathological records collected from 1961 to 1999 at the Nagasaki University Hospital [15]. The estimated doses in Nagasaki survivors who were not shielded at the time of explosion were 924.7 centigrays (cGy) at 1 km and 120.7 cGy at 1.5 km from the hypocenter [21]. Simultaneously, we have already analyzed *HER2 *and *C-MYC *gene amplification by FISH method with FFPE samples and revealed that 26 out of 35 cases show clear hybridization signals for *HER2 *and/or *C-MYC *gene amplification. In this study, 15 (mean age: 58.0 years, range: 45.4-82.8 years) out of 26 cases are available for aCGH analysis because there is a limit to the amount of tissues. As control subjects, 13 cases of invasive ductal carcinoma from calendar year-matched patients (matched on date of both diagnosis and birth; mean age: 55.5 years, range: 43.0-69.1 years), who did not receive "Health Handbook" according to the Atomic Bomb Survivors' Medical Treatment Law, were also analyzed. All clinicopathologic information including exposure distance, diagnosis, the modified Bloom-Richardson histologic grading, had been determined in our previous study [15]. Clinicopathological findings of these samples are provided in Additional file 1, Table S1. All experimental procedures for this study were approved by Committee for the Ethical Issues on Human Genome and Gene Analysis at Nagasaki University (Protocol No. 0305150036-2).

### DNA extraction

Tumor DNA was extracted from FFPE archival tissues, as reported previously [22]. Briefly, using ten 10 μm-thick sections, tumor areas containing more than 70% tumor cells, identified by a guide slide stained with hematoxylin and eosin, were microdissected from each FFPE block. Paraffin removal was performed in 80% xylene and tissues were washed twice with absolute ethanol, and deparaffinized tissue pieces were spun down. After drying, pellets were resuspended in 360 μL of buffer ATL (QIAmp DNA Mini Kit, Quiagen, Germany) and incubated at 95°C for 15 minutes, followed by cooling to room temperature. Samples were immediately digested with proteinase K for three days at 56°C in a rotation oven with periodic mixing and addition of fresh proteinase K every 24 hours. DNA was collected using the QIAmp DNA Mini Kit according to the manufacturer's instructions. Specifically, 400 μL of buffer AL (equal volume to sample suspension) was added to the sample and incubated at 70°C for 10 minutes. 400 μL of absolute ethanol was then added. The sample solution was then placed into the spin column and centrifuged for 1 minute at 8000 × g. The spin column was washed twice with 500 μL of buffer AW1 by centrifugation at 8000 × g for one minute and then washed with buffer AW2 by centrifugation at 14,000 × g for three minutes. The DNA was finally eluted with 55 μL buffer AE. Extracted DNA was quantified on a NanoDrop ND-1000 spectrophotometer (NanoDrop Technologies, Wilmington, DE, USA).

### aCGH analysis

The Genomic DNA ULS Labeling Kit (Agilent technologies, USA) was used to chemically label 500 ng of tumor DNA from samples and from reference female genomic DNA (Promega, USA) with Cy5 or Cy3 dye for 30 minutes at 85°C, respectively, followed by purification using Agilent-KREA*pure*™ columns. Because ULS method labeled DNA with fluorescent dyes directly without any amplification steps or enzymatic reaction, this method is suitable for aCGH analysis using degraded DNA such as from FFPE blocks [18-20]. Dye-flip analyses were conducted on 6 of 28 samples, where samples were labeled with Cy3 and references were labeled with Cy5. Purified, labeled samples were then combined and mixed with human Cot-1 DNA (Invitrogen, USA), Agilent 10× Blocking Agent and Agilent 2× Hybridization Solution. Prior to array hybridization, hybridization mixtures were denatured at 95°C for 3 minutes and incubated at 37°C for 30 minutes. Agilent CGH*block *was added and samples were hybridized to the SurePrint G3 Human CGH 8 × 60 K Microarray, which contains 8 identical arrays consisting of ~63,000 in situ synthesized 60-mer oligonucleotide probes that span coding and noncoding sequences with an average spatial resolution of ~54 kb. Hybridization was carried out at 65°C for 40 hours before washing in Agilent Oligo aCGH Wash Buffer 1 at room temperature for 5 minutes, followed by washing in Agilent Oligo aCGH Wash Buffer 2 at 37°C for 1 minute.

Scanning and image analysis were done on an Agilent DNA Microarray Scanner. Feature Extraction Software (version 9.5) was used for data extraction from raw microarray image files. Agilent Genomic Workbench (version 5.0) was used to visualize, detect and analyze chromosomal patterns using an ADM-2 algorithm with the threshold set to 5.5. A copy number gain was defined as a log 2 ratio > 0.25 and a copy number loss was defined as a log 2 ratio < -0.25.

### Statistical analysis

The total length of the CNA, which is the sum of each segment gained or lost, was used as an indicator of GIN. To determine the effect of each clinicopathological factor on the natural logarithm of GIN, Student's (Welch's) t-test or analysis of variance and the significance test of Pearson's correlation coefficient were performed. Means and proportions of each clinicopathological factor were compared between A-bomb survivors and control using t-tests, Fishers exact tests and Cochran-Armitage tests. We evaluated the impact of A-bomb exposure, age at the time of diagnosis, storage time, histological grade according to the modified Bloom-Richardson histologic grading system [23], derivative log ratio spread (DLRSpread), which estimates the log ratio noise by calculating the spread of log ratio differences between consecutive probes along all chromosomes, *HER2 *amplification and *C-MYC *amplification determined by FISH on GIN using analysis of covariance which is a technique that combines the features of analysis of variance and regression. Our model was

$$Y_{ij}=\mu_{i}+\overset{6}{\underset{k=1}{\sum }}\beta_{k}\left(X_{kij}-{\bar{X}}_{k..}\right)+\varepsilon_{ij}$$

where *Y_ij _*is the natural logarithm of GIN of the *j*th observation in the *i*th class and *μ_i _*represents the population means of the A-bomb exposure classes, *β_k _*is the regression coefficient of *Y *on *X_k_*, *ε_ij _*is the residual. Here, *X_k _*is the variable which represents age at the time of diagnosis, DLRSpread, *HER2 *amplification, *C-MYC *amplification, histological grade and storage time.

Effects were considered statistically significant when P-values were less than 0.05. The CORR, TTEST, FREQ and GLM procedures in the SAS system (version 9.1.3) was utilized for calculation.

## Results

### Results of aCGH analysis

The mean of the DLRSpread was 0.54 (range, 0.26 to 1.05) (Additional file 1, Table S1). As a quality assessment measure, we examined the concordance of the dye-flip analysis and the correlation between aCGH and FISH results concerning *HER2 *and *C-MYC *oncogene amplification. In the 6 samples with dye-flip analyses, the mean of the concordance rate of each paired sample was 76.0% (range, 43.2% to 96.1%) (Additional file 2, Table S2). The concordance rate of each paired sample was defined as the ratio of length of copy number aberrant region in one dye combination to the dye-flipped combination in each sample. To confirm the validity of aCGH results using FFPE samples, we compared the results of amplification status of *HER2 *and *C-MYC *in the aCGH and FISH results. *HER2 *was amplified in 9 of 25 samples, in which showed clear hybridization signals in the FISH analysis. In 7 of these 9 samples, the log 2 ratio for the probe sets (A_14_P121276, A_14_P114826 and triplicate of A_16_P20643178) corresponding to the *HER-2 *gene was > 0.25, which met our criteria for a gain based on aCGH results. The sensitivity, specificity and overall accuracy for the *HER2 *gene were 77.8%, 93.8% and 88%, respectively (Additional file 3, Figure S1). Whereas *C-MYC *was amplified in 11 of 23 samples, in which showed clear hybridization signals in the FISH analysis, only two of these 11 samples showed a gain for the probe sets (A_14_P128991, A_14_P138867 and A_14_P137636) corresponding to the *C-MYC *gene based on aCGH results. The sensitivity, specificity and overall accuracy for the *C-MYC *gene were 18%, 75% and 48%, respectively (Additional file 4, Figure S2).

In our detection setting, the ADM-2 algorithm with the threshold set to 5.5, CNA were detected in all samples. The mean of the total number of site and the length of CNA were 10.29 (range, 1 to 28) and 105,400,874 bp (range, 607,921 bp to 525,839,497), respectively (Additional file 1, Table S1), and these values varied from case to case (Additional file 5, Figure S3).

### Correlation between GIN and clinicopathological findings

The results of comparisons of clinicopathological profiles of breast cancer between A-bomb survivors and control are shown in Table 1. Proportions of histological grade and the incidence of HER2 amplification were significantly higher in A-bomb survivors than in controls (P = 0.04, P = 0.04, respectively), which is consistent with our data published previously [15]. The total length and number of CNA tended to be larger in the A-bomb survivors (P = 0.15, P = 0.16, respectively).

**Table 1:** Comparisons of clinicopathological factors of breast cancers between A-bomb survivor and control.

Clinicopathological profile	A-bomb survivors (n = 15)	Control (n = 13)	*P-*value
Mean age of onset (years old)	58.0 (52.6, 63.4)^†^	55.5 (49.7, 61.4)	0.51^1)^
Mean tumor size (cm)	24.7 (20.7, 28.8)	36.2 (20.2, 52.3)	0.15^1)^
Histological subtype			
Papillo-tubular	9	4	0.29^2)^
Solid-tubular	1	2	
Scirrhous	5	7	
Histological grade			
I	1	3	0.04^3)^
II	5	7	
III	9	3	
Lymph node metastasis			
Positive	8	5	0.60^4)^
Negative	4	4	
Unknown	3	4	
ER status			
Positive	7	8	0.26^4)^
Negative	8	5	
PgR status			
Positive	7	8	0.43^4)^
Negative	8	5	
*HER2 *amplification (FISH)			
Positive	7	2	0.04^4)^
Negative	5	11	
No signal	3	0	
*C-MYC *amplification (FISH)			
Positive	9	2	0.09^4)^
Negative	5	7	
No signal	1	4	
Mean total length of CNA (bp)	64,032,415 (29,443,979, 139,238,660)	23,924,175 (6,936,445, 82,515,771)	0.15^1)^
Mean number of CNA	12.2 (8.4, 16.0)	8.08 (3.0, 13.1)	0.16^1)^

†: 95% confidence interval, CNA: copy number aberrations

1): t-test, 2): χ^2^-test, 3): Cochran-Armitage test, 4): Fisher's exact test

The correlations between the total length of CNA and histological subtypes, histological grade, status of axillary lymph node metastasis, status of estrogen receptor (ER), *HER2/C-MYC *amplifications determined by FISH, age at the time of diagnosis, tumor size, age of samples, DLRSpread, age of the time at the A-bomb exposure, the exposure distance from the hypocenter and time between age at diagnosis and age at exposure were tested (Table 2, Table 3). Among these factors, DLRSpread was negatively correlated with the total length of CNA significantly (P = 0.034, r = -0.40) and age at the time of diagnosis, age of samples tended to be correlated with that negatively (P = 0.055, r = -0.37) and positively (P = 0.064, r = 0.35), respectively. Notably, among A-bomb survivors, latent period from irradiation was inversely correlated with the total length of CNA, indicating an involvement of GIN in the case of breast cancer which showed early onset from an initiation event by A-bomb exposure.

**Table 2:** Comparisons of total length of copy number aberrations (CNA) by clinicopathological factor of breast cancers.

Clinicopathological factor	Total (N = 28) n (%)	Mean total length of CNA (bp)	*P-*value
Histological subtype			
Papillo-tubular	13 (46)	27,487,678	0.54^1)^
Solid-tubular	3 (11)	41,158,092	
Scirrhous	12 (43)	61,520,542	
Histological grade			
I	4 (14)	49,011,523	0.32^2)^
II	12 (43)	47,158,730	
III	12 (43)	32,711,871	
Axillary lymph node metastasis			
Positive	13 (62)	30,993,870	0.30^3)^
Negative	8 (38)	59,602,019	
ER status			
Positive	14 (50)	53,249,555	0.43^3)^
Negative	14 (50)	30,863,969	
*HER2 *amplification (FISH)			
Positive	9 (36)	28,970,829	0.75^3)^
Negative	16 (64)	37,017,451	
*C-MYC *amplification (FISH)			
Positive	11 (48)	38,698,059	0.46^3)^
Negative	12 (52)	22,102,472	

1): analysis of variance, 2): Jonckheere-Terpstra trend test, 3): t-test

**Table 3:** Correlation analyses between clinicopathological factors and total length of copy number aberrations in breast cancers.

	Mean total length of copy number aberrations (bp)			
	
Clinical factors	All cases (n = 28)		A-bomb survivors (n = 15)	
	r*	*P*-value*	r*	*P*-value*

Age at the time of diagnosis	-0.37	0.055	-0.59	0.021
Tumor size (cm)	0.042	0.83	-0.25	0.37
Storage time (years)	0.35	0.064	0.49	0.067
DLRSpread	-0.40	0.034	-0.38	0.16
Age of the time of exposure to the A-bomb**			-0.31	0.25
Exposure distance from the hypocenter (km)**			0.11	0.70
Time between age at diagnosis and exposure (year)**			-0.52	0.047

*Pearson's correlation coefficient. **Only among A-bomb survivors

**Figure 1:**
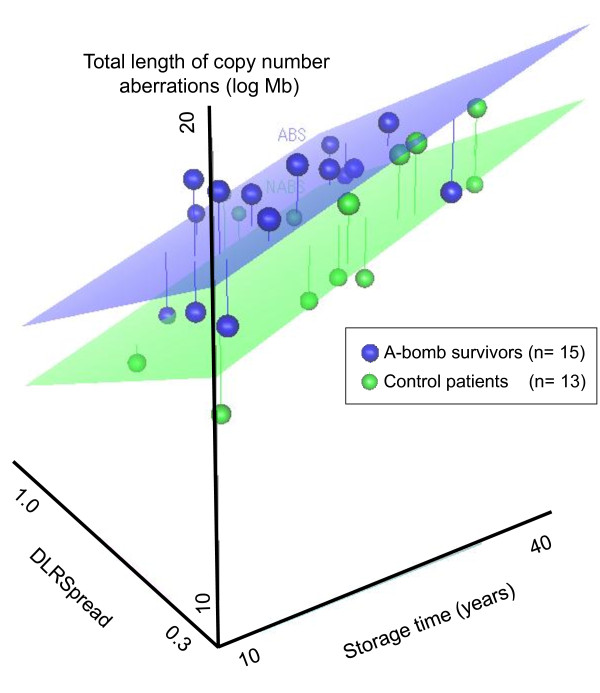
**Relationship between genomic instability and affecting clinical factors**. Blue and green plots indicate atomic bomb survivors and control patients, respectively. Blue and Green plane represent regression plane of each group. X-axis; years of sample storage time. Y-axis; derivative log ratio spread (DLRSpread). Z-axis; natural logarithm of total number of copy number aberrations.

The multivariate analysis using analysis of covariance revealed that the status of A-bomb exposure was the most significant factor for the total length of CNA even excluding the effect of *HER2 *and *C-MYC *amplification, histological grade, age at the time of diagnosis, age of samples and DLRSpread (Figure 1, Table 4). Analysis of covariance-adjusted difference in means between the A-bomb exposed group and the unexposed group is 63,151,697 (95%CI, 18,291,298 to 151,682,068; P = 0.005) for GIN.

**Table 4:** Multivariate analyses with covariance in total length of copy number aberrations in breast cancers.

Source of Variation	DF*	Mean Squares	*F*-value	*P-*value*
A-bomb exposure	1	20.59	11.62	0.005
HER2 amplification (FISH)	1	0.65	0.37	0.556
C-MYC amplification (FISH)	1	4.14	2.34	0.152
Histological Grade	1	1.06	0.60	0.454
Age at the time of diagnosis	1	2.35	1.32	0.272
Storage time (years)	1	8.78	4.96	0.046
DLRSpread	1	3.77	2.13	0.170

*Degrees of Freedom

## Discussion

Ionizing radiation is an established risk factor for breast cancers [24-27]. Several epidemiologic reports have suggested that an increased risk of cancer has continued for decades after exposure, and that a higher risk of certain types of cancers still persists in A-bomb survivors [9-14]. Thus, a long-lasting health effect is considered to be a contributing factor in tumorigenesis in A-bomb survivors. We have recently demonstrated an association of oncogene amplification in breast cancers among A-bomb survivors with radiation exposure [15], which can be regarded as being the results of positive selection during breast carcinogenesis. This finding suggests that A-bomb radiation may affect the development of oncogene amplification by inducing a higher level of GIN in breast cancers found in survivors. The current study was carried out to further confirm the enhanced GIN in A-bomb radiation-associated breast cancers using the aCGH method. The aCGH method is a quite useful technique to detect the DNA CNA as an indicator of GIN, which represents chromosomal loss and gain caused by radiation-induced DNA double-strand breaks [16]. Unger et al. [28] found DNA CNA pattern which is characteristic of radiation-induced papillary thyroid cancer in residents living in the vicinity of Chernobyl using the aCGH method.

Tissue samples from A-bomb survivors are considered to be extremely valuable biological materials with which to analyze the radiation signature or radiation-associated human health effects, particularly in low-dose and late exposures. The molecular analyses of carcinogenesis in A-bomb survivors require clinical data of individuals and biological materials with pathologic data of tumors. Our database, which consist of two independent databases: a clinical database providing exposure distance on Nagasaki survivors registered at our institute which was established in 1972 and a pathological database by the Nagasaki Tumor Tissue Registry (NTTR) which was established in 1974, allow us to obtain FFPE archival tissue samples resected from A-bomb survivors. For the genomic analyses, we confirmed the utility of FFPE archival tissue with FISH methods to detect gene amplification despite DNA degradation caused by fixation and long storage. In the present study, we conducted an aCGH analysis using tumor DNA extracted from FFPE archival breast cancer samples from A-bomb survivors. To our knowledge, this is the first attempt to perform an aCGH analysis with solid tumors from A-bomb survivors. The samples used in this study were very old, with ranges 14 to 43 years (with a mean of 25 years) in storage. The DLRSpread obtained was 0.26 to 1.05, with a mean of 0.54, which indicated the relatively lower quality of this experiment compared with that expected with DNA from fresh frozen tissue or peripheral blood lymphocyte. However, the status of *HER2 *oncogene amplification based on aCGH result was highly concordant with the results of FISH that the sensitivity, specificity and accuracy were 77.8%, 93.8% and 88%, respectively, which were comparable to the results from former aCGH studies with FFPE archival tissue [29,30]. By contrast, the concordance was low for the status of *C-MYC *oncogene amplification between the results from aCGH and FISH, with the sensitivity, specificity and accuracy being 18%, 75% and 48%, respectively. This discordance, especially in sensitivity, may result from the use of only three probes on the *C-MYC *gene and a smaller change in amplification at the region including *C-MYC *than the *HER2 *gene. Our results suggest that the 60K×8 CGH array is a reliable technology to identify gene copy number aberration with definite changes.

Our aCGH analysis showed a great deal of variety in its amount and pattern of genomic alterations from case to case. In comparison with previous reports on breast cancers from general population, mean number of CNA in our cases seemed to be relatively small (mean: 12.2, range: 2-28) but recurrently affected regions (8q24.3, 17q12, 19p13.11, 1q21.2-q22: Additional file 5, Figure S3) found in our cases were concordant [4,7,31-33]. However, direct comparisons of the current results with published results in aCGH are practically difficult because the results of aCGH analyses are greatly influenced by the array design and type of samples (e.g., fresh frozen or FFPE). A previous study of an aCGH analysis of radiation-induced and spontaneous rat mammary carcinoma indicated that the frequency of carcinoma having any CNA and the number of CNA in radiation-induced carcinoma were significantly greater than that observed in the spontaneous carcinoma [34]. Another study of an aCGH analysis of premenopausal breast cancers in the residents from a nuclear fallout-contaminated area in Belarus did not show any significant differences or tendencies in the average number of total DNA CNA compared with matched control cases from Western New York, even though breast cancer from Belarus had significantly more average number of gains [35]. These discrepancies may result from differences in the experimental models, since the former is a study of a simplified animal cancer model and the latter is an observational study of human cancer affected by many etiological factors. But the present study endorsed the former result with a tendency for breast cancer in A-bomb survivors to have a higher number of CNA (P = 0.16, Table 1, Additional file 1, Table S1). Furthermore, mean total length of CNA were also larger, if not significantly, in the A-bomb survivors than control group (P = 0.15, Table 1, Additional file 1, Table S1). Herein, we assumed the total length of CNA as an indicator of GIN because the amount of CNA represents the consequences of double-strand breaks, abnormal DNA damage repairs and gross rearrangements of chromosomes [1,16], and a consecutive changes of probes is considered to be much more important than a change of only one probe in such experimental model using high density probes and relatively noisy data. Since high histological grade, ER negative expression, early age of onset and *HER2 *amplification were reported to be correlated with higher incidence of genomic aberrations [4], we examined the correlation between the total length of CNA and clinicopathological factors, followed by multivariate analysis using analysis of covariance to evaluate the impact (effect) of A-bomb exposure, age at the time of diagnosis, HER2 and C-MYC amplification, histological grade, storage time, and DLRSpread on GIN, which have shown that the status of A-bomb exposure showed a significant correlation after the exclusion of confounding factor by the multivariate analysis (Table 4). Thus, we have demonstrated that breast cancers in A-bomb survivors harbored significant GIN independently of the effect of other clinicopathological factors.

## Conclusions

The present study indicated that archival FFPE tissues from A-bomb survivors are useful for genome-wide aCGH analysis and A-bomb radiation exposure induced GIN not only at the region of the *HER2 *and *C-MYC *oncogenes but throughout the whole genome in breast cancers by aCGH. The crucial mechanisms that can account for the continuously higher incidence of breast cancers in A-bomb survivors for decades remain to be determined. Further research on the molecular mechanisms to induce a long-lasting GIN in the breast tissue from survivors can contribute to an understanding of radiation-associated carcinogenesis.

## Competing interests

The authors declare that they have no competing interests.

## Authors' contributions

MO participated in the design of the study and carried out aCGH analysis. KY participated in the design of the study. HK performed the statistical analysis. SM conducted pathological analysis. TN participated in the design of the study. MN conceived of the study and participated in the design of the study. All authors read and approved the final manuscript.

## Supplementary Material

Additional file 1 **Table S1. Summary of clinicopathological factors and aCGH Analysis**. Click here for file

Additional file 2 **Table S2. Result of dye-flip analysis**. Click here for file

Additional file 3 **Figure S1. Chromosomal view of chromosome 17 and comparison of the results from FISH and aCGH analyses on *HER2 *oncogene**. Log2 ratio values for all oligonucleotide probes are plotted as a function of their chromosomal position. Each point represents a single probe and the blue vertical line indicates the position of the *HER2 *oncogene. Aberration calls identified by ADM-2 algorithm are shown. Click here for file

Additional file 4 **Figure S2. Chromosomal view of chromosome 8 and comparison of the results from FISH and aCGH analyses on *C-MYC *ongcogene**. Log2 ratio values for all oligonucleotide probes are plotted as a function of their chromosomal position. Each point represents a single probe and the blue vertical line indicates the position of the *C-MYC *oncogene. Aberration calls identified by ADM-2 algorithm are shown. Click here for file

Additional file 5 **Figure S3. Graphic display of whole genomic aberrations in atomic bomb survivors (upper panel) and control patients (lower panel)**. The panels to the right of each chromosome shows the frequency of gains, indicated by the red bars ranging from 0% to 100%, and losses, indicated by the green bars ranging from 0% to 100%. Click here for file
