# Supplementary material for: Significance of genomic instability in breast cancer in atomic bomb survivors: analysis of microarray-comparative genomic hybridization
Source: Radiat Oncol. 2011 Dec 7;6:168. doi: 10.1186/1748-717X-6-168 (PMC3280193; doi:10.1186/1748-717X-6-168)
Supplement: Additional file 2 — Table S2. Result of dye-flip analysis. [file 1748-717X-6-168-S2.DOC]

| **Table S2** Result of Dye-flip Analysis | | | | | |
| --- | --- | --- | --- | --- | --- |
| ID | Total length of chromosomal aberrant region (bp) | | | | Overlap(ratio) |
|  | Exam 1* | Exam 2* | Mean | Overlap (size) |  |
| E1 | 935,074,531 | 871,475,239 | 903,274,885 | 853,783,220 | 94.5% |
| E3 | 87,812,674 | 187,565,702 | 137,689,188 | 75,266,738 | 54.7% |
| E4 | 87,434,730 | 70,514,371 | 78,974,551 | 66,717,274 | 84.5% |
| C1 | 27,525,342 | 25,439,712 | 26,482,527 | 25,439,717 | 96.1% |
| C2 | 82,380,041 | 115,613,024 | 98,996,533 | 82,380,042 | 83.2% |
| C3 | 27,657,722 | 14,417,777 | 21,037,750 | 9,083,925 | 43.2% |
| Mean | 207,980,840 | 214,170970 | 211,075,905 | 185,445,153 | 76.0% |

*Exam 1: sample-Cy5, reference-Cy3 *Exam 2: sample-Cy3, reference-Cy5
