# Supplementary figures and images for: Significance of genomic instability in breast cancer in atomic bomb survivors: analysis of microarray-comparative genomic hybridization
Source: Radiat Oncol. 2011 Dec 7;6:168. doi: 10.1186/1748-717X-6-168 (PMC3280193; doi:10.1186/1748-717X-6-168)

## Slide 1
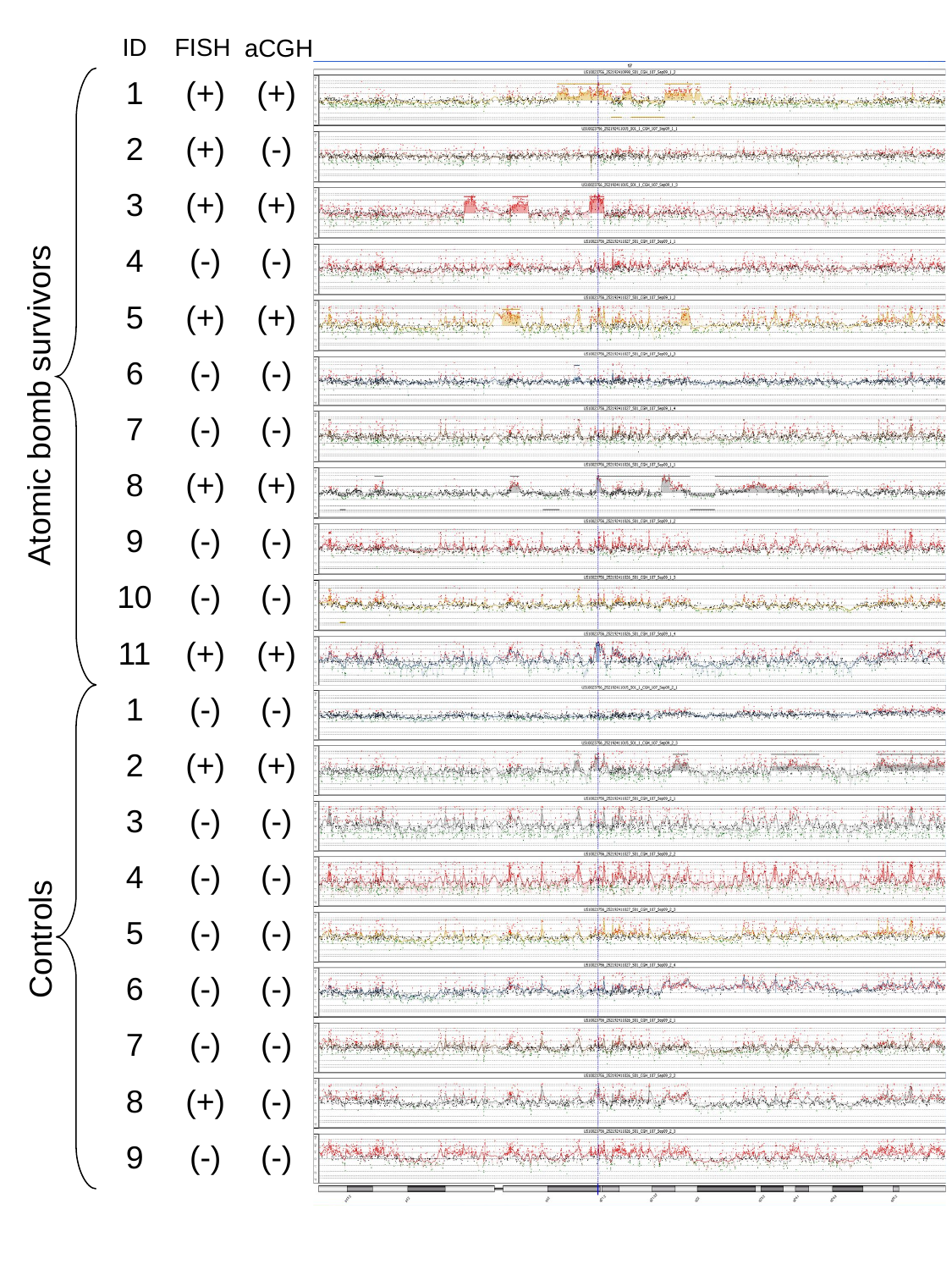

ID
FISH
aCGH
| 1 | (+) | (+) |
| --- | --- | --- |
| 2 | (+) | (-) |
| 3 | (+) | (+) |
| 4 | (-) | (-) |
| 5 | (+) | (+) |
| 6 | (-) | (-) |
| 7 | (-) | (-) |
| 8 | (+) | (+) |
| 9 | (-) | (-) |
| 10 | (-) | (-) |
| 11 | (+) | (+) |
| 1 | (-) | (-) |
| 2 | (+) | (+) |
| 3 | (-) | (-) |
| 4 | (-) | (-) |
| 5 | (-) | (-) |
| 6 | (-) | (-) |
| 7 | (-) | (-) |
| 8 | (+) | (-) |
| 9 | (-) | (-) |
Atomic bomb survivors
Controls

Supplement: Additional file 3 — Figure S1. Chromosomal view of chromosome 17 and comparison of the results from FISH and aCGH analyses on HER2 oncogene. Log2 ratio values for all oligonucleotide probes are plotted as a function of their chromosomal position. Each point represents a single probe and the blue vertical line indicates the position of the HER2 oncogene. Aberration calls identified by ADM-2 algorithm are shown. [file 1748-717X-6-168-S3.PPT]

## Slide 1
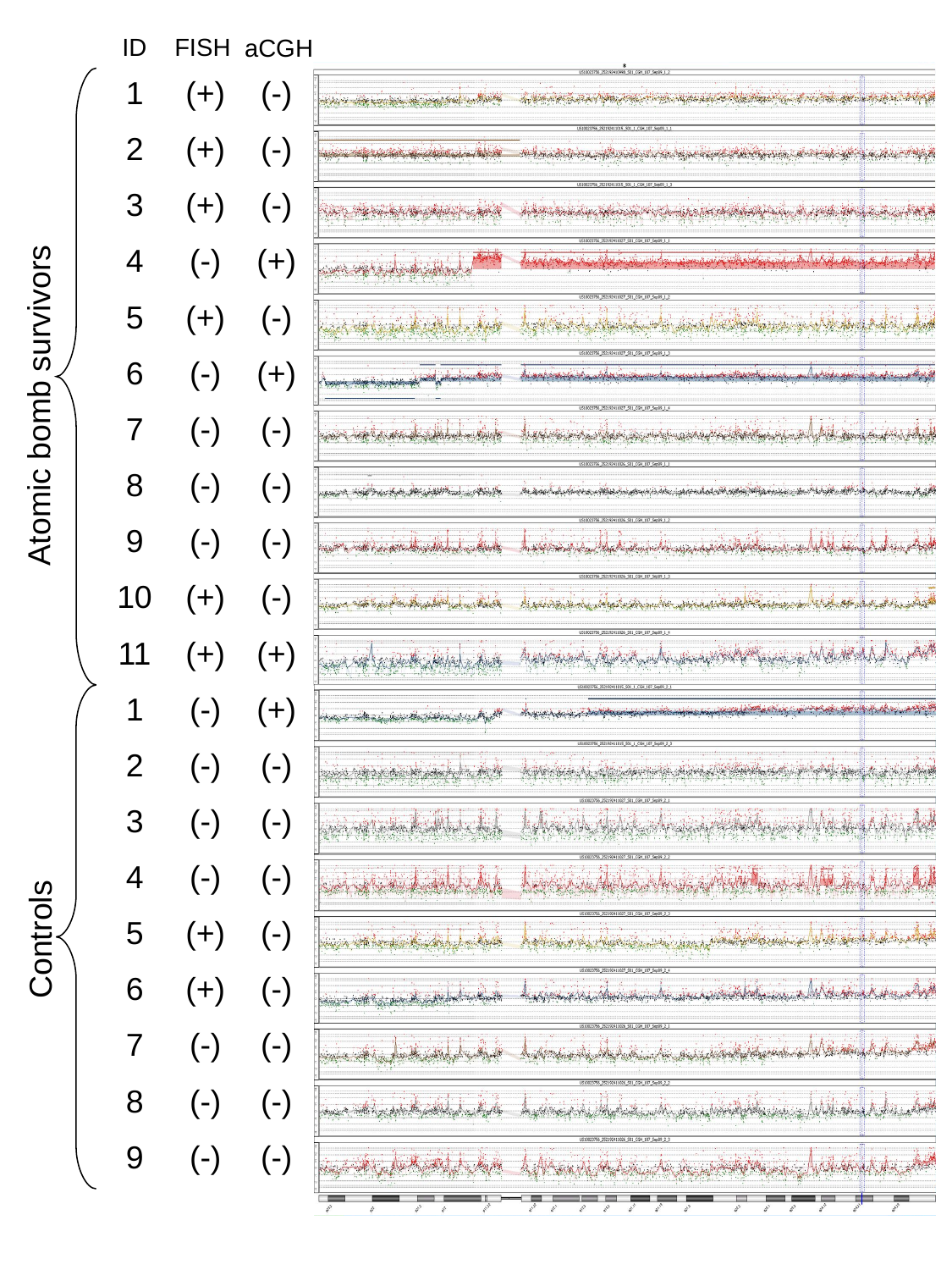

ID
FISH
aCGH
| 1 | (+) | (-) |
| --- | --- | --- |
| 2 | (+) | (-) |
| 3 | (+) | (-) |
| 4 | (-) | (+) |
| 5 | (+) | (-) |
| 6 | (-) | (+) |
| 7 | (-) | (-) |
| 8 | (-) | (-) |
| 9 | (-) | (-) |
| 10 | (+) | (-) |
| 11 | (+) | (+) |
| 1 | (-) | (+) |
| 2 | (-) | (-) |
| 3 | (-) | (-) |
| 4 | (-) | (-) |
| 5 | (+) | (-) |
| 6 | (+) | (-) |
| 7 | (-) | (-) |
| 8 | (-) | (-) |
| 9 | (-) | (-) |
Atomic bomb survivors
Controls

Supplement: Additional file 4 — Figure S2. Chromosomal view of chromosome 8 and comparison of the results from FISH and aCGH analyses on C-MYC ongcogene. Log2 ratio values for all oligonucleotide probes are plotted as a function of their chromosomal position. Each point represents a single probe and the blue vertical line indicates the position of the C-MYC oncogene. Aberration calls identified by ADM-2 algorithm are shown. [file 1748-717X-6-168-S4.PPT]

## Slide 1
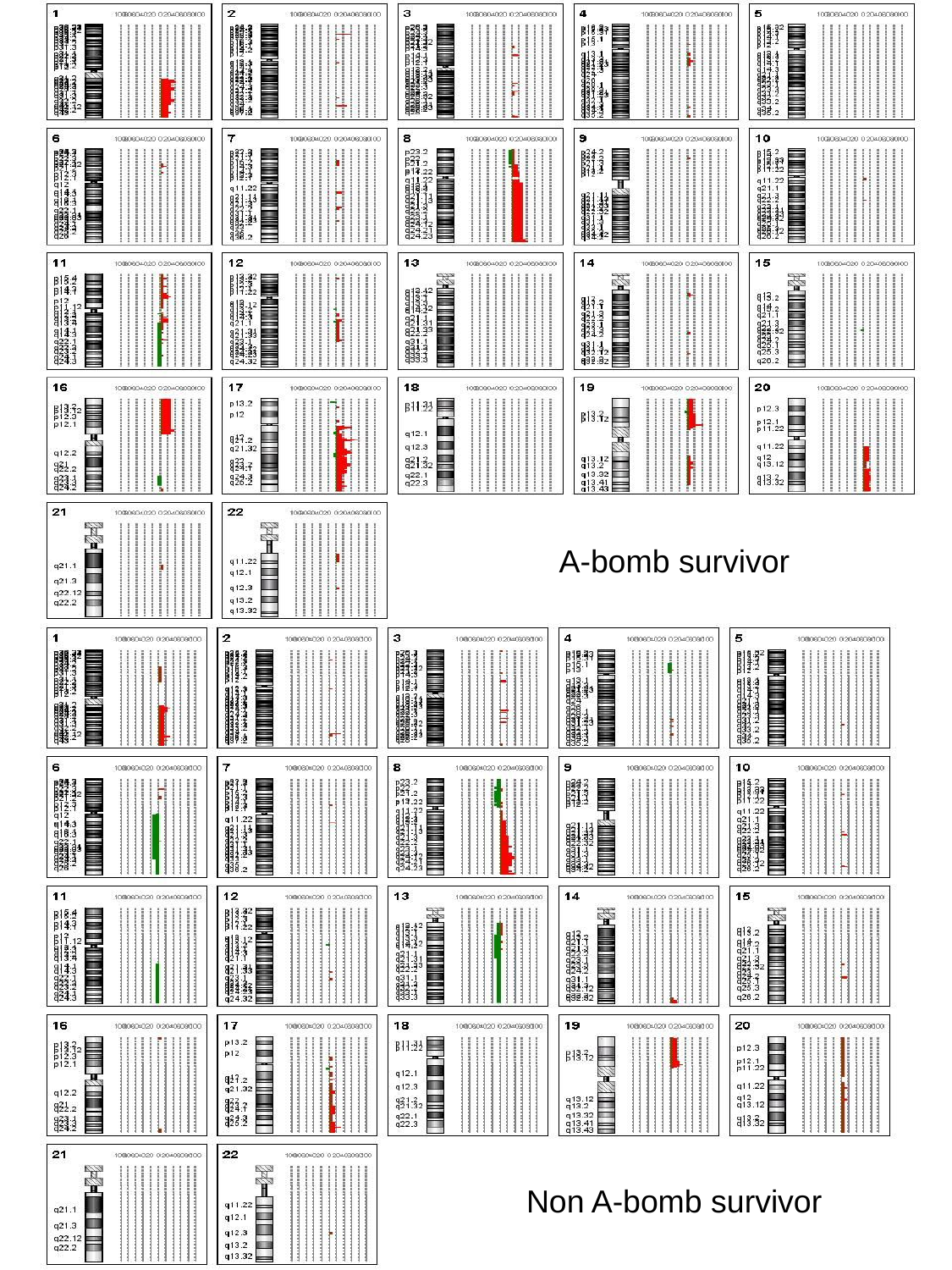

A-bomb survivor
Non A-bomb survivor

Supplement: Additional file 5 — Figure S3. Graphic display of whole genomic aberrations in atomic bomb survivors (upper panel) and control patients (lower panel). The panels to the right of each chromosome shows the frequency of gains, indicated by the red bars ranging from 0% to 100%, and losses, indicated by the green bars ranging from 0% to 100%. [file 1748-717X-6-168-S5.PPT]
